# Author Correction: Human blood type influences the host-seeking behavior and fecundity of the Asian malaria vector *Anopheles stephensi*

**DOI:** 10.1038/s41598-022-06705-7

**Published:** 2022-02-10

**Authors:** Shahmshad Ahmed Khan, Nur Faeza Abu Kassim, Cameron Ewart Webb, Muhammad Anjum Aqueel, Saboor Ahmad, Sadia Malik, Taimoor Hussain

**Affiliations:** 1 Department of Entomology, University College of Agriculture, University of Sargodha, Sargodha, Pakistan; 2 Department of Entomology, Pir Mehr Ali Shah Arid Agriculture University Rawalpindi, Rawalpindi, Pakistan; 3 129 Medical Entomology Laboratory, School of Biological Sciences, Universiti Sains Malaysia, 11800 Minden, Penang Malaysia; 4 Marie Bashir Institute for Infectious Diseases and Biosecurity, University of Sydney, Sydney, New South Wales Australia; 5 Department of Entomology, Faculty of Agriculture & Environment, The Islamia University of Bahawalpur, Bahawalpur, Pakistan; 6 Key Laboratory of Pollinating Insect Biology, Ministry of Agriculture, Institute of Apicultural Research, Chinese Academy of Agricultural Sciences, Beijing, 100081 China; 7 Fatima Jinnah Women University, Rawalpindi, Pakistan; 8 Department of Agronomy, Pir Mehr Ali Shah Arid Agriculture University Rawalpindi, Rawalpindi, Pakistan

Correction to: *Scientific Reports* https://doi.org/10.1038/s41598-021-03765-z, published online 21 December 2021

In the original version of this Article, Shahmshad Ahmed Khan was incorrectly listed as a corresponding author. The correct corresponding author for this Article is Nur Faeza Abu Kassim. Correspondence and request for materials should be addressed to nurfaeza@usm.my.

Muhammad Anjum Aqueel was incorrectly affiliated with ‘Department of Entomology, University College of Agriculture, University of Sargodha, Sargodha, Pakistan’ and ‘Department of Entomology, UCA & ES, The Islamia University of Bahawalpur, Bahawalpur, Pakistan’. The correct affiliation is listed below.

Department of Entomology, Faculty of Agriculture & Environment, The Islamia University of Bahawalpur, Bahawalpur, Pakistan.

The original Article has been corrected.

